# Expansion of a chromosomal repeat in *Escherichia coli*: roles of replication, repair, and recombination functions

**DOI:** 10.1186/1471-2199-10-14

**Published:** 2009-02-23

**Authors:** Anthony R Poteete

**Affiliations:** 1 Department of Molecular Genetics and Microbiology, University of Massachusetts Medical School, Worcester, MA, USA

## Abstract

**Background:**

Previous studies of gene amplification in *Escherichia coli *have suggested that it occurs in two steps: duplication and expansion. Expansion is thought to result from homologous recombination between the repeated segments created by duplication. To explore the mechanism of expansion, a 7 kbp duplication in the chromosome containing a leaky mutant version of the *lac *operon was constructed, and its expansion into an amplified array was studied.

**Results:**

Under selection for *lac *function, colonies bearing multiple copies of the mutant *lac *operon appeared at a constant rate of approximately 4 to 5 per million cells plated per day, on days two through seven after plating. Expansion was not seen in a *recA *strain; null mutations in *recBCD *and *ruvC *reduced the rate 100- and 10-fold, respectively; a *ruvC recG *double mutant reduced the rate 1000-fold. Expansion occurred at an increased rate in cells lacking *dam*, *polA, rnhA*, or *uvrD *functions. Null mutations of various other cellular recombination, repair, and stress response genes had little effect upon expansion. The *red *recombination genes of phage lambda could substitute for *recBCD *in mediating expansion. In the *red*-substituted cells, expansion was only partially dependent upon *recA *function.

**Conclusion:**

These observations are consistent with the idea that the expansion step of gene amplification is closely related, mechanistically, to interchromosomal homologous recombination events. They additionally provide support for recently described models of RecA-independent Red-mediated recombination at replication forks.

## Background

Expression of a chromosomal gene in *Escherichia coli *can be elevated by gene amplification. The mechanism of this amplification is thought to consist of two steps, duplication and expansion. Duplication is rare, largely *recA*-independent, and occurs between microhomologies in the chromosome as a replication accident. Expansion is frequent, *recA*-dependent, and thought to result from unequal crossing-over events between the duplicated segments [1-3].

Recent investigations of gene amplification in *E. coli *have focused on amplification of plasmid-borne genes. A phenotypically leaky F'-borne mutation, *ϕ(lacIX13-lacZ)*, gives rise to Lac^+ ^revertants bearing amplified arrays of 40–80 copies of the *lac *region [4]. Lac^+ ^revertants of F'*lac *bearing the +1 frameshift allele *ϕ(lacI33-lacZ)*, extensively employed in studies of adaptive mutation, consist mainly of one-base deletions in runs of iterated bases [5,6], but clones bearing amplified arrays appear at a lower rate as well [7,8]. Properties of *lac *amplification have generally supported the duplication-expansion model. (i) An engineered duplication of the frameshift mutant *lac *locus amplifies at a greatly elevated frequency [9], as predicted by the idea that duplication is the rate-limiting step (and as had been seen in the case of chromosomal *ampC *[2]). (ii) Amplification is dependent upon *recBCD *and *ruvABC*, as well as *recA*, indicating an important role for homologous recombination [10].

Expansion of a pre-existing repeat has also been studied primarily on plasmids. In one study, a pBR322 derivative was constructed with two directly repeated *tetA *genes, each bearing an inactivating mutation, but arranged in such a way that a single unequal crossover would generate an array of three copies, one of which was a functioning gene. In this system, expansion was reduced only five-fold in a *recA *mutant; expansion was elevated in strains bearing mutations in *dnaQ*, *dnaE*, *dnaB*, or *dnaN *[11].

Expansion of a pre-existing duplication was compared with amplification of a single copy of F'-borne *ϕ(lacI33-lacZ) *in another study [10]. Expansion was found to be increased in a *polA *mutant, and unaffected by overexpression of *xonA*, while amplification from a single copy was inhibited by both of these conditions. It was concluded that the amplification defects caused by the *polA *mutant and by *xonA *overproduction were in duplication, not expansion.

**Figure 1:**
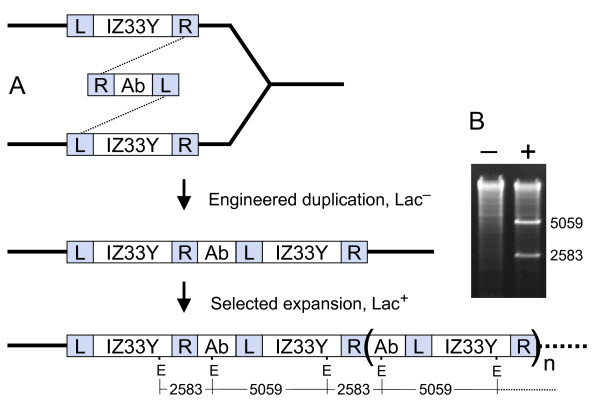
**Expansion of a chromosomal duplication**. A. Chromosomal *(lacI33-lacZ)-lacY *[38] was duplicated by phage λ Red-mediated recombination with a linear DNA bearing homology-flanked antibiotic resistance marker Ab. A hypothetical mechanism by which the duplication could be generated, involving crossovers between the linear DNA and both copies of the replicating chromosomal target, is diagrammed [9]. The duplication was constructed with a tetracycline resistance element, which was later replaced with *cat*. Under selection for Lac function, the *(lacIZ33Y)*_2_-*cat *duplication expands into multiple copies. L and R – chromosomal sequences flanking *lac*. E – EcoR1 restriction sites. B. Multiple copies of the repeated sequence are seen as bands produced by EcoR1 digestion of cellular DNA. Tests of two Lac^+ ^revertants, one without (-), and one with (+) an expanded *lac *array, are shown as examples.

This study was undertaken to characterize expansion of a repeated sequence in the bacterial chromosome. A duplication of chromosomal *ϕ(lacI33-lacZ) *was constructed (Fig. 1). As expected, it expands at a high rate under selection for function. The effects of mutations in various recombination, replication, DNA repair, and stress response genes on expansion of the duplication were tested. The findings support the idea that expansion occurs via homologous recombination, and suggest as well that many of the recombination events leading to expansion take place at replication forks.

## Results

**Figure 2:**
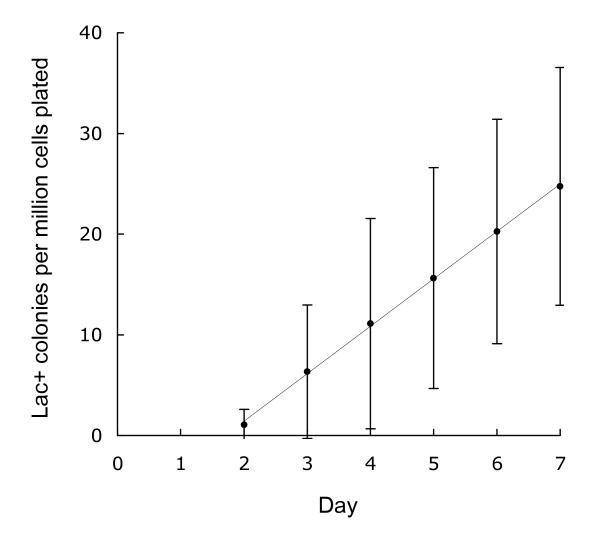
**Kinetics of Lac^+ ^colony formation**. Cultures of TP1004, an MG1655 derivative bearing the *(lacIZ33Y)*_2_-*cat *duplication, were plated on lactose minimal agar. Data points represent mean daily colony counts from 19 independent cultures. Error bars represent 1 standard deviation. A least-squares linear regression curve is shown; its slope is 4.71 colonies per million viable chloramphenicol-resistant cells plated per day.

An *E. coli *strain bearing a chromosomal duplication of the leaky *ϕ(lacI33-lacZ) *allele, when plated on minimal medium containing lactose as the only available carbon source, gives rise to approximately 1000-fold more colonies, over the course of a week, than an otherwise isogenic strain bearing a single copy. As shown in Fig. 2, the colonies start appearing two days after plating, and accumulate at an average rate of approximately 4–5 per million viable duplication-bearing cells plated per day, two to seven days after plating. Daily colony counts vary widely between independent cultures, as well as day-to-day on the same plate. This variation is considerably greater than that observed in experiments with single copy F'-borne *ϕ(lacI33-lacZ)*, in which the appearance of colonies after day two fits a Poisson distribution, implying that the mutations occurred after plating [12]. In contrast, the variation in colony counts seen in experiments with the chromosomal duplication strain indicate that most of the variation between cultures exists prior to plating (unpublished data). This observation is consistent with the hypothesis that each culture contains copy number variants which arise during growth, and that the probability of colony formation varies with copy number at the time of plating. Despite this variability, if 12 or more independent cultures are plated, and daily colony counts are averaged, the rate of accumulation is seen to be nearly constant, as reflected in the close fit of the data points to a straight line.

The Lac^+ ^colonies appearing in these experiments can arise either by expansion or by mutation. A strain bearing even a single chromosomal copy of the un-frameshifted *lacI-lacZ *fusion grows well on lactose minimal medium. However, most or all of the excess Lac^+ ^colonies produced by the duplication-bearing strain contain expanded arrays of the structure diagrammed in Fig. 1. The amplified sequences in these clones are readily visualized as specific bands in restriction enzyme digests of total cellular DNA [4]. In tests of 28 Lac^+ ^revertants from 22 cultures bearing the duplication, including four colonies which appeared on day two, all had expanded; none of 10 single-copy revertants contained amplified arrays (Table 1). Quantitation of total DNA, and DNA in the amplified bands, as described in the methods section, from 10 Lac^+ ^clones, indicated a mean *lac *copy number of 72, with a standard deviation of 17. Expansion of this magnitude would be expected to result in β-galactosidase production comparable to a single-copy un-frameshifted gene, as the frameshift mutation reduces β-galactosidase production 100-fold [12]. The modest variability of *lac *copy number in the Lac^+ ^clones presumably reflects a sort of optimum or equilibrium, in which the benefit of more β-galactosidase is balanced by the cost of extra DNA in the chromosome (70 copies of the repeat make the chromosome roughly 10% larger). Cultures of the expanded-array variants contain unknown numbers of point-revertant *lac *genes. Under continued selection for *lac *function, it is possible that the population would eventually be taken over by revertants which, bearing a single good copy of *lac*, have the benefit of sufficient β-galactosidase without the cost of more DNA. However, it is likely that such a changeover would take many generations because the Lac^+ ^revertant has, if anything, only a small selective advantage – it does not have a noticeably faster growth rate on lactose minimal medium, for example. Hastings et al. [8] tested this idea, and found that amplified *ϕ(lacI33-lacZ) *clones kept under selection for *lac *function do not form revertants readily.

**Table 1:** Expansion and survival tests

Genotype^a^	Lac^+ ^clone expansion test No. positive/no. tested	Survival on lactose minimal medium relative to wild type^b^
wild type	28/28	1
recA	0/10	0.9 ± 0.1
recBCD	9/10	0.9 ± 0.1
ruvC	10/10	1.2 ± 0.4
recG	18/18	nd
ruvC recG	6/14	0.6 ± 0.2
red+	10/10	nd
red+ recA	8/10	nd
wild type single copy	0/10	nd

a. See the legend to Figure 4 for a description of the strains tested for expansion. For the survival test, the *(lacIZ33Y)*_2_-*cat *duplication was replaced by a non-reverting *lac *deletion.

b. Means and standard errors are shown for three measurements. nd = not determined.

**Figure 3:**
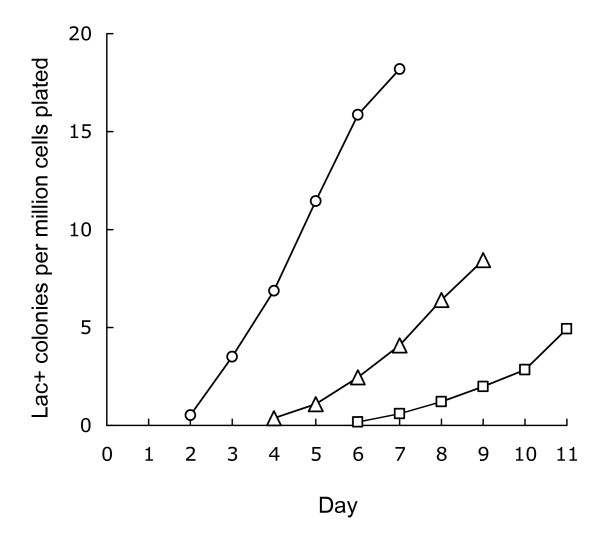
**Adaptive nature of the expansion**. Cultures of TP1004, an MG1655 derivative bearing the *(lacIZ33Y)*_2_-*cat *duplication, were plated on minimal agar in which lactose was made available as the only available carbon source, either at the time of plating (circles), or after two (triangles) or four (squares) days of incubation. Data points represent mean daily colony counts from 12 independent cultures. Error bars are omitted for clarity; as in Fig. 2, the standard deviations are comparable in magnitude to the means.

The clones appearing as Lac^+ ^colonies acquire their ability to grow on lactose while under selection. Colonies re-streaked on lactose minimal plates form colonies visible to the unaided eye in 24 hours or less, regardless of whether they were picked on day 2 or day 7. Reversion and amplification of F'-borne *ϕ(lacI33-lacZ) *are adaptive, in that they occur only in the presence of lactose, not when the bacteria are simply starved [8,13]. The leakiness of the mutant allele is critical for adaptive mutation: residual lactose metabolism is enough to power the replication/recombination/repair processes involved, though not enough for cell division [12]. The experiment graphed in Fig. 3 shows that expansion of a chromosomal *ϕ(lacI33-lacZ) *is similarly adaptive. Cultures were plated on minimal medium containing no available carbon source. Lactose was added after two or four days by injection under the agar slab. There was no sudden burst of colonies appearing two or three days later, as would be expected if expanded *lac *arrays had accumulated during starvation. Rather, the kinetics of appearance of Lac^+ ^colonies resembled that seen in the cells initially plated on lactose, with a delay of either two or four days, and at declining rates, suggesting that the starving cells gradually lost their potential to expand.

**Figure 4:**
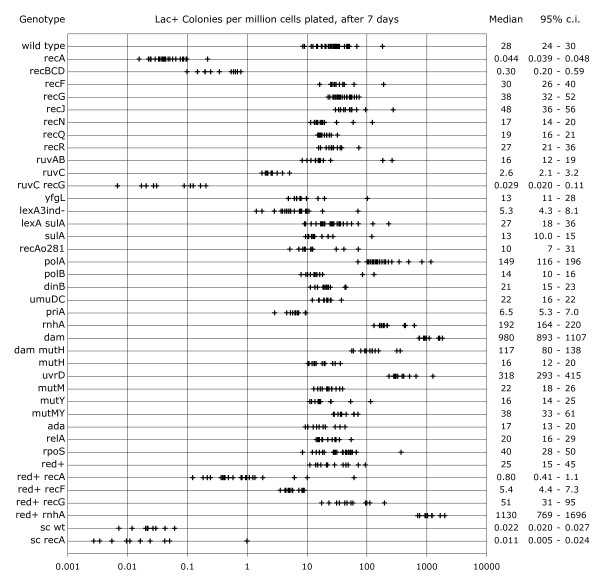
**Roles of replication, repair, and recombination functions in expansion**. Multiple independent cultures of the indicated genotype were plated on lactose minimal medium. Strains are all MG1655 derivatives. All except the ones labeled "sc" (for single copy) bear the the *(lacIZ33Y)*_2_-*cat *duplication. Strains labeled "red+" bear the phage λ *red *recombination genes, which replace the *recC-ptr-recB-recD *gene cluster in the *E. coli *chromosome. Except for the *red *substitution, the *lexA *alleles, and the *recAo281 *operator mutation, all the alleles are nulls made by substituting an antibiotic resistance element for the coding sequence of the gene.

Strains combining the duplication with mutations in DNA transaction genes were tested for Lac^+ ^colony formation (Fig. 4). A null mutation in *recA *reduced the rate nearly 1000-fold, nearly down to that of a strain bearing a single copy of *ϕ(lacI33-lacZ) *(labeled "sc" near the bottom of Fig. 4). The *recA *mutation had no significant effect in the single copy background. The strong *recA *dependence of expansion in this experiment contrasts with the weak *recA *dependence seen in a previous study of expansion of a plasmid-borne duplication [11], but is not surprising, for two reasons: (i) The duplicated segment in this study was much larger, and *recA *dependence tends to increase with increasing homology lengths [14,15]. (ii) The assay employed in the previous study required only a single recombination event, whereas becoming strongly Lac^+ ^by expansion of a chromosomal *ϕ(lacI33-lacZ) *duplication probably involves more than one recombination event.

Null mutations in *recBCD *and *ruvC *reduced Lac^+ ^colony formation 100-fold and 10-fold, respectively. Other mutations eliminating single recombination functions, *recF*, *recG*, *recN*, *recQ*, *recR*, and *ruvAB*, had little overall effect. A *ruvC recG *double mutant was also tested. Like the *recA *mutation, and as in other homologous recombination events [16], it generated Lac^+ ^colonies approximately 1000-fold less efficiently than wild type.

A disruption of the *E. coli yfgL *gene was reported to confer a strong recombination/repair deficiency phenotype [17]. As shown in Fig. 4, however, a *yfgL *null mutation constructed for this study has little or no effect on expansion. It also confers no UV-sensitivity or transductional recombination phenotype, in either an MG1655 or an AB1157 strain background (not shown); others have found no recombination/repair phenotype associated with a *yfgL *null as well [18].

To test the hypothesis that the deficiencies of *recA*, *recBCD*, *ruvC*, and *ruvC recG *mutants in Lac^+ ^colony formation are due to their inability to expand the duplication, two alternative explanations were considered and ruled out. (i) Lac^+ ^revertants of these mutants could grow much more slowly than Lac^+ ^revertants of wild type. Lac^+ ^colonies were restreaked on minimal lactose plates on the days they arose. In the cases of wild type, *recA*, *recBCD*, and *ruvC*, all of the Lac^+ ^clones formed colonies visible to the unaided eye by 24 hours after restreaking, independent of the day on which they arose. In the case of the *ruvC recG *double mutant, none of the 8 tested Lac^+ ^revertants formed visible colonies by 24 hours, but all did so by 48 hours. However, counting the *ruvC recG *colonies after 8 days instead of 7 only increased the median from 0.029 to 0.056 colonies per million viable cells plated (data not shown). Thus, slow growth of revertants can account for only a small part of the deficiencies of the recombination mutants in forming Lac^+ ^colonies. (ii) The mutants could be proficient at expansion, but deficient at survival on the selection plates. Survival of the mutants on lactose minimal medium was tested as described in the methods section. The results (Table 1) indicate that none of the mutants has a substantial survival defect relative to wild type.

The Lac^+ ^revertants of the deficient mutants consist of varying populations of expanded and mutated clones (Table 1). In the case of *recA*, none of 10 tested clones had an expanded *lac *array. The frequency of expanded arrays among *recBCD *revertants was 9 out of 10; *ruvC *was 10 out of 10, *recG *was 18 out of 18, and *ruvC recG *was 6 out of 14.

The Lac^+ ^reversion phenotype of a *recG *null mutant is more complex than the data in Fig. 4 suggest. The mutant strain's rate of colony formation tends to increase sharply late in the experiment, with the new colonies tending to appear as satellites of older colonies (not shown). Factors influencing the timing and extent of this satellite-based population explosion include plating density, but are otherwise unknown. The *recG *mutant data shown in Fig. 4 are from selected experiments, in which the plating density was low, and satellitism was not as strongly evident as in other experiments. Satellitism of this sort suggests that something produced by the older colonies on the plate stimulates recombination, perhaps via a genotoxic effect. It is consistent with the finding that overexpression of *recG *protects *E. coli *against weak organic acids [19].

The effects on expansion of varying RecA activity were tested by plating mutants affecting *recA *expression. The uninducible *lexA3 *mutation has been reported to reduce the frequency of a number of different homologous recombination events [20]; it also reduced Lac^+ ^colony formation approximately five-fold. The SOS-constitutive *lexA71::Tn5 *mutation (in a *sulA *null background, to suppress its lethality) had no significant effect; neither did the *recAo281 *operator constitutive allele.

A number of replication genes were tested for roles in expansion. A null mutation in *polA *increased the rate of expansion 5-fold; null mutations in the other non-essential DNA polymerase-encoding genes *polB*, *dinB*, and *umuDC*, had little or no effect. Loss of the replication restart function *priA *caused a small decrease in expansion efficiency, while loss of *rnhA *caused a nearly 7-fold increase.

A strain lacking *dam *function exhibited a 35-fold elevated rate of Lac^+ ^colony formation. Apparently, part of this elevated rate is due to the double-strand breaks which occur as the result of mis-directed mismatch repair in *dam *mutants [21,22]. As shown in Fig. 4, a *dam mutH *double mutant exhibited an intermediate rate between those of wild type and the *dam *single mutant. The *mutH *null allele by itself had little or no effect.

Other DNA repair functions were tested for effects on expansion as well. A *uvrD *null mutant formed Lac^+ ^colonies at a 10-fold elevated rate, while *mutM*, *mutY*, a *mutM mutY *double, and an *ada *null mutation had no significant effects.

The question of whether expansion of a chromosomal repeat occurs as part of a stress response, like amplification starting from a single episomal copy [23], was explored by testing null mutations in *rpoS *and *relA*. As shown in Fig. 4, these mutations had little or no effect on Lac^+ ^colony formation.

Expansion mediated by the Red recombination system of phage λ was studied in a series of strains in which the phage *red *genes replace the *recC-ptr-recB-recD *gene cluster in the *E. coli *chromosome (designated "red+" in Fig. 4). Replacing RecBCD with Red has no effect on the rate of Lac^+ ^colony formation, but it changes the extent to which expansion is dependent upon other recombination functions. In the *red*-substituted background, a *recA *null mutation reduces Lac^+ ^colony formation only 35-fold. Among the *recA *revertants, 8 of 10 that were tested contained expanded *lac *arrays (Table 1), showing that Red, unlike RecBCD, can promote expansion in the absence of RecA. Red-mediated expansion is reduced by a *recF *null mutation, and elevated slightly by a *recG *null; these mutant effects are seen in Red-mediated gene replacement events as well [24]. The *rnhA *null mutation has a stronger effect in the *red*-substituted background than in wild type, elevating the rate of expansion 45-fold.

## Discussion and conclusion

The genetic requirements of homologous recombination in *E. coli *vary with the particular event examined, but some features of chromosomal events are nearly general: dependence on *recA *and *recBCD*, and mild or no dependence upon a variety of other recombination functions whose roles are revealed mainly in the absence of *recBCD *function [14]. Expansion by duplicated chromosomal *ϕ(lacI33-lacZ) *fits this general pattern. Similarly, mutations with known hyper-rec phenotypes – *polA*, *dam*, *uvrD*, and *rnhA *[20,25,26] – also cause an elevated rate of expansion. These observations support the idea that expansion is best understood as a homologous recombination event or series of events.

The Red recombination system of phage λ promotes RecA-independent recombination between chromosomes if at least one of the chromosomes is replicating, and RecA-dependent recombination between non-replicating chromosomes [27-30]. The RecA-independent expansion seen in *red*-substituted bacteria suggests that at least some of the Red-mediated recombination events involved in expansion take place at replication forks [30,31].

The involvement of replication in recombination events leading to expansion is additionally suggested by the increased rates of expansion of the *rnhA *and *dam *mutants. Replication in both these mutants escapes cell cycle regulation. In an *rnhA *mutant, unsynchronized DNA replication initiates at multiple sites in the chromosome [32]. In a *dam *mutant, initiation is confined to *oriC *but is not regulated [33]. Elevated recombination frequencies in both of these mutants may be due, at least in part, to an increased occurrence of double strand breaks. Both exhibit greatly reduced viability in the absence of RecBCD function, possibly because RecBCD is needed to repair the excess double strand breaks [34,35]. Eliminating the mismatch repair endonuclease MutH in a *dam *mutant prevents the double strand breaks which result from mis-directed mismatch repair, but does not bring expansion down to the wild type level; unregulated replication itself is a possible cause of the residual excess expansion in the *dam mutH *double mutant. The mechanism by which improperly regulated replication forks provoke more recombination events than normally regulated replication forks is unknown, but there are a number of possible explanations. They might be more prone to breaking down or to running into each other, or just more numerous in the cell.

## Methods

### Duplication of ϕ(lacI33-lacZ)-lacY

Plasmid pTP1029 [36] is a vector containing the *tetRA *genes from Tn10 and the *pir*-dependent replication origin R6K*origamma*. pTP1060 was made by ligating a synthetic DNA made from two oligodeoxyribonucleotides, GATCCAGGTTCTTTGAGCTCTTTGGCGGCCGC and GATCGCGGCCGCCAAAGAGCTCAAAGAACCTG, into the *Bam*HI site of pTP1029. pTP1016 [36] contains *E. coli *sequences which normally flank the *lacI *and *lacY *genes; in the plasmid, they flank the *cat *gene. pTP1027 [36] and pTP1049 [37] contain the same flank sequences as pTP1016, with wild type *lacIZY *and *ϕ*(*lacI33-lacZ)-lacY*, respectively, between them. pTP1061 was made by ligating the *cat*-with-*lac*-flanks cassette of pTP1016 into the NotI site of pTP1060. A strain bearing a duplication of the chromosomal segment containing *ϕ*(*lacI33-lacZ)-lacY *was constructed by electroporating strain TP890 with *Bsr*G1- and *Sph*1-digested plasmid pTP1061, and selecting for tetracycline resistance (see Fig. 1). The *tet-origamma *insert between the duplicated *lac *copies was replaced by *cat *via recombination with a linear DNA generated by PCR with a Tn9-containing strain as template, and primers GATCCCGCGGAATAACATCATTTGGTGACGAAATAACTAAATGAGACGTTGATCGGCACG and CCACGATGCGTCCGGCGTAGAGGATCTGAAGATCAGCAGTATTCAGGCGTAGCACCAGGC. The presence of duplicated segments in bacterial chromosomes was verified by the use of PCR with divergent primers, as described [36]. Other strain construction details are given in Tables 2 and 3.

**Table 2:** Strains

Strain	Relevant Genotype	Reference, source, or construction
FC691	lacIZ33^a^	[38]
GM3819	damΔkan	M. Marinus
GM8291	polAΔfrt-kan/F' polA+ camR	M. Marinus; polA allele [39]
MG1655	wild type	
MV1132	srl300::Tn10 recAo281	M. Volkert
MV1154	lexA3	M. Volkert
MV2104	lexA71::Tn5	M. Volkert
TP538	recGΔtet	[40]
TP539	recGΔkan	[40]
TP540	ruvABΔtet	[40]
TP547	red-cat^b^	derivative of KM32 [41]
TP577	recFΔtet	[40]
TP605	sulAΔtet	[40]
TP643	recQΔtet	[24]
TP645	recRΔtet	[24]
TP662	sulAΔkan	substitution of Tn903 aph for Tn10 tetRA in sulAΔtet [40]
TP664	recNΔtet	[42]
TP730	red-cat lacIZ33	FC691 × P1•TP547
TP732	red-pae-cI^b ^lacIZ33	TP730 × pTP822 linear [41]
TP796	recAΔtet	[43]
TP797	ruvCΔtet	[43]
TP798	red-cat	[36]
TP832	red-amp^b^	[36]
TP838	recBCDΔtet	[43]
TP872	red-amp lacΔcat	TP832 × cat15,16 pcr of Tn9^c^
TP889	lacΔcat	MG1655 × P1•TP872
TP922	red-pae-cI^b ^(lacIZ33Y)_2_-tet-oriR6Kã^d^	TP732 × pTP1061 linear^e^
TP929	(lacIZ33Y)_2_-tet-oriR6Kgamma	TP889 × P1•TP922
TP942	red-pae-cI (lacIZ33Y)_2_-cat	TP922 × Toc1,2 pcr of Tn9
TP997	galK::aacC1067	[44]
TP1000	dinBΔtet	TP798 × din7,8 pcr of Tn10
TP1003	lacΔspc	TP889 was transduced with a P1 lysate of an unnamed intermediate strain, TP798 × LAT2,3 pcr of Tn21 aadA
TP1004	(lacIZ33Y)_2_-cat	TP929 × P1•TP942
TP1005	(lacIZ33Y)_2_-cat dinBΔtet	TP1004 × P1•TP1000
TP1006	(lacIZ33Y)_2_-cat recAΔtet	TP1004 × P1•TP796
TP1007	(lacIZ33Y)_2_-cat recFΔtet	TP1004 × P1•TP577
TP1008	(lacIZ33Y)_2_-cat recGΔtet	TP1004 × P1•TP538
TP1009	(lacIZ33Y)_2_-cat recNΔtet	TP1004 × P1•TP664
TP1011	(lacIZ33Y)_2_-cat recQΔtet	TP1004 × P1•TP643
TP1012	(lacIZ33Y)_2_-cat recBCDΔtet	TP1004 × P1•TP838
TP1014	(lacIZ33Y)_2_-cat ruvABΔtet	TP1004 × P1•TP540
TP1015	(lacIZ33Y)_2_-cat ruvCΔtet	TP1004 × P1•TP797
TP1020	(lacIZ33Y)_2_-cat recRΔtet	TP1004 × P1•TP645
TP1022	lacIZ33	Spontaneous chloramphenicol-sensitive TP1004 derivative
TP1031	red-cat recJΔtet	TP798 × recJ1,2 pcr of Tn10
TP1032	red-cat rhnAΔtet	TP798 × rnhA1,2 pcr of Tn10
TP1033	red-cat polBΔtet	TP798 × polB1,2 pcr of Tn10
TP1034	(lacIZ33Y)_2_-cat recJΔtet	TP1004 × P1•TP1031
TP1035	(lacIZ33Y)_2_-cat rhnAΔtet	TP1004 × P1•TP1032
TP1036	(lacIZ33Y)_2_-cat polBΔtet	TP1004 × P1•TP1033
TP1038	red-amp (lacIZ33Y)_2_-cat	TP832 × P1•TP942
TP1042	(lacIZ33Y)_2_-cat damΔkan	TP1004 × P1•GM3819
TP1043	(lacIZ33Y)_2_-cat sulAΔtet	TP1004 × P1•TP605
TP1047	(lacIZ33Y)_2_-cat uvrDΔtet	TP1004/pKM208 × uvrD1,2 pcr of Tn10
TP1048	(lacIZ33Y)_2_-cat sulAΔtet lexA71::Tn5	TP1043 × P1•MV2104
TP1049	(lacIZ33Y)_2_-cat umuDCΔtet	TP1004/pKM208 [45] × umDC1,2 pcr of Tn10
TP1050	(lacIZ33Y)_2_-cat malFΔspc	TP1004/pKM208 × malF1,2 pcr of TP997
TP1051	(lacIZ33Y)_2_-cat rpoSΔtet	TP1004/pKM208 × rpoS1,2 pcr of Tn10
TP1053	(lacIZ33Y)_2_-cat adaΔkan	TP1004/pKM208 × ADA1,2 pcr of Tn903 aph
TP1054	(lacIZ33Y)_2_-cat priAΔkan	TP1004/pKM208 × priA1,2 pcr of Tn903 aph
TP1055	(lacIZ33Y)_2_-cat relAΔkan	TP1004/pKM208 × relA1,2 pcr of Tn903 aph
TP1056	(lacIZ33Y)_2_-cat sulAΔtet priAΔkan	TP1043 × P1•1054
TP1057	red-amp (lacIZ33Y)_2_-cat recAΔtet	TP1038 × P1•796
TP1058	red-amp (lacIZ33Y)_2_-cat recFΔtet	TP1038 × P1•577
TP1059	red-amp (lacIZ33Y)_2_-cat recGΔtet	TP1038 × P1•538
TP1061	red-amp (lacIZ33Y)_2_-cat rnhAΔtet	TP1038 × P1•1035
TP1062	lacIZ33 recAΔtet	TP1022 × P1•796
TP1063	(lacIZ33Y)_2_-cat recAΔkan	TP1004/pKM208 × recA3,4 pcr of Tn903 aph
TP1064	(lacIZ33Y)_2_-cat mutHΔtet	TP1004/pKM208 × mutH1,2 pcr of 1016
TP1065	(lacIZ33Y)_2_-cat mutMΔtet	TP1004/pKM208 × mutM1,2 pcr of Tn10
TP1066	(lacIZ33Y)_2_-cat mutYΔkan	TP1004/pKM208 × mutY1,2 pcr of Tn903 aph
TP1069	(lacIZ33Y)_2_-cat mutMΔtet mutYΔkan	TP1065 × P1TP1066
TP1080	lacΔcat recAΔtet	TP889 × P1•TP796
TP1081	lacΔcat recBCDΔtet	TP889 × P1•TP838
TP1082	lacΔcat ruvCΔtet	TP889 × P1•TP797
TP1086	(lacIZ33Y)_2_-cat polAΔfrt-kan	TP1004 × P1•JW3835
TP1089	(lacIZ33Y)_2_-cat yfgLΔkan	TP1004/pKM208 × yfgL1,2 pcr of Tn903 aph
TP1090	(lacIZ33Y)_2_-cat lexA3	TP1050 × P1•MV1154, selection for Mal^+^, screen for UV-sensitivity
TP1091	(lacIZ33Y)_2_-cat damΔkan mutHΔtet	TP1064 × P1•GM3819
TP1095	(lacIZ33Y)_2_-cat srl300::Tn10 recAo281	TP1063 × P1•MV1132
TP1098	(lacIZ33Y)_2_-cat ruvCΔtet recGΔkan	TP1015 × P1•TP539
TP1099	(lacIZ33Y)_2_-cat recAo281	TP1095 × P1•TP1063, selection for Srl+, screen for kanamycin sensitivity and the Sph site created by the recAo281 mutation [46]

Notes

a. The abbreviation *lacIZ33 *is used to denote the triple mutant *ϕ(lacI33-lacZ) *bearing the *lacI*^*Q *^promoter-up mutation in the *lacI *promoter, a 212-bp deletion fusing *lacI *to *lacZ*, and a +1 frameshift mutation in the lacI sequences near the fusion junction [13].

b. Strains designated "red" bear the phage λ *red *recombination genes, which replace the *recC-ptr-recB-recD *gene cluster in the *E. coli *chromosome.

c. Primers used in polymerase chain reactions to generate antibiotic cassettes for gene replacements are described in Table 3.

d. Strains designated "*(lacIZ33Y)*_2_-" bear a duplication of *lacIZ33 *and *lacY*, with the genetic element listed after the hyphen (*tet-oriR6Kgamma *or *cat*) inserted between the two copies.

e. Details of this construction are given in the text.

**Table 3:** Primers

Primer	Sequence
ADA1	GATTATGAAAAAAGCCACATGCTTAACTGACGATCAACGCACGTTGTGTCTCAAAATCTC
ADA2	CTCCTCATTTTCAGCTTCGCGGCGCAGCAGTTGCGCTTTACAACCAATTAACCAATTCTG
cat15	TCTGGTGGCCGGAAGGCGAAGCGGCATGCATTTACGTTGAATGAGACGTTGATCGGCACG
cat16	AGAGTACATCTCGCCGTTTTTTCTCAATTCATGGTGTACAATTCAGGCGTAGCACCAGGC
din7	GCTGGATAAGCAGCAGGTGCTTTCGCAGCGAACGCGTTAACTCGACATCTTGGTTACCGT
din8	ACCAGTTGTCTTTCCATTTGCGGGTCAAGCAACGTCACATCGCGGAATAACATCATTTGG
LAT2	AAGAAAGCCTGACTGGCGGTTAAATTGCCAACGCTTATTATTATTTGCCGACTACCTTGG
LAT3	GACGGGTTGTTACTCGCTCACATTTAATGTTGATGAAAGCAAACGGATGAAGGCACGAA
malF1	GTCCTGGAATGAGGAAGAACCCCATGGATGTCATTAAAAAAAACGGATGAAGGCACGAA
malF2	CCCTTAATCAAACTTCATTCGCGTGGCTTTCAGGTTCACTTTATTTGCCGACTACCTTGG
mutH1	TTTTTTAATCAAGGTATCATGACATGTCCCAACCTCGCCCCTCGACATCTTGGTTACCGT
mutH2	GCGATGGCTACTGGATCAGAAAATGACGGGCCAGTAGTGCCGCGGAATAACATCATTTGG
mutM1	GCATCTGTTCATTCCTGGAGATGCTATGCCTGAATTACCCCTCGACATCTTGGTTACCGT
mutM2	TCCGGCGCGCATGAATTACTTCTGGCACTGCCGACAATAACGCGGAATAACATCATTTGG
mutY1	CAACAGTGAATTCGGTGACCATGCAAGCGTCGCAATTTTCACGTTGTGTCTCAAAATCTC
mutY2	CTTTATCGACTCACGCGCTAAACCGGCGCGCCAGTGCGTACAACCAATTAACCAATTCTG
polB1	GTGGCGCAGGCAGGTTTTATCTTAACCCGACACTGGCGGGCTCGACATCTTGGTTACCGT
polB2	AGTGGTGAACGTTGGTAGTCCAGCGGCTCCGGGCCGTTGGCGCGGAATAACATCATTTGG
priA1	GATGCTATGCCCGTTGCCCACGTTGCCTTGCCCGTTCCGCACGTTGTGTCTCAAAATCTC
priA2	GGAATCCGGTATTGTATTGATGAGCGCCAGCGTACCGTTACAACCAATTAACCAATTCTG
recA3	TTACCCGGCATGACAGGAGTAAAAATGGCTATCGACGAACACGTTGTGTCTCAAAATCTC
recA4	CCCTTGTGTATCAAACAAGACGATTAAAAATCTTCGTTTACAACCAATTAACCAATTCTG
recJ1	ACAGATACAACTTCGTCGCCGTGAAGTCGATGAAACGGCACTCGACATCTTGGTTACCGT
recJ2	GCAGTGGACCGCCGCCGACCGGTTCGACCATCACCTTCAACGCGGAATAACATCATTTGG
relA1	GGACGATGGTTGCGGTAAGAAGTGCACATATCAATAAGGCACGTTGTGTCTCAAAATCTC
relA2	GGTCATGTCGATGGTCGCCAGTTGCTGTTTGGTGTCGCTACAACCAATTAACCAATTCTG
rnhA1	GGCAATCCAGGACCTGGGGGTTACGGCGCTATTTTACGCTCTCGACATCTTGGTTACCGT
rnhA2	GCCGCGGCACGAGCCAGTTCATCACAGCGTTCGTTTTCCGCGCGGAATAACATCATTTGG
rpoS1	CGGGTAGGAGCCACCTTATGAGTCAGAATACGCTGAAAGTCTCGACATCTTGGTTACCGT
rpoS2	CCTTTCTGACAGATGCTTACTTACTCGCGGAACAGCGCTTCGCGGAATAACATCATTTGG
Toc1	GATCCCGCGGAATAACATCATTTGGTGACGAAATAACTAAATGAGACGTTGATCGGCACG
Toc2	CCACGATGCGTCCGGCGTAGAGGATCTGAAGATCAGCAGTATTCAGGCGTAGCACCAGGC
umDC1	CAGATTATTATGTTGTTTATCAAGCCTGCGGATCTCCGCGCTCGACATCTTGGTTACCGT
umDC2	CCTGCCGCTATATTTATTTGACCCTCAGTAAATCAGAACTCGCGGAATAACATCATTTGG
uvrD1	AACCTATTTTTACGCGGCGGTGCCAATGGACGTTTCTTACCTCGACATCTTGGTTACCGT
uvrD2	CTGGCCCTGAAATGCCACCTGCAAACGGCTATGCTCACCGCGCGGAATAACATCATTTGG
yfgL1	TCTGAGAGGGACCCGATGCAATTGCGTAAATTACTGCTGCACGTTGTGTCTCAAAATCTC
yfgL2	GCCGTCAGCGGCAACCGGTTCAGTCTGGAAACCGGAACTACAACCAATTAACCAATTCTG

### Plating methods

Strains to be tested for reversion to Lac^+ ^were grown to saturation in M9 0.1% glycerol minimal medium at 37°C, and plated on M9 0.1% lactose plates at 37°C. M9 minimal media, supplemented with thiamine at 5 μg/ml, were as described [7]. In most cases, viable duplication-positive titers were determined by plating on LB agar supplemented with chloramphenicol at 10 μg/ml, which permits colony formation only by bacteria retaining the *cat *gene between the duplicated segments. Strains with poor viability in rich media (*ruvC recG, polA, priA*) were titered on M9 glucose plates; retention of the duplication in these cases was assessed by testing the chloramphenicol resistance of individual colonies from the titer plates. Lactose minimal plates were inoculated with 1–2 × 10^9 ^cells, either of the strain to be tested by itself, or, in most cases, of the strain to be tested plus a non-reverting, *lac *deletion-bearing scavenger strain [13]. The rates of appearance of Lac^+ ^colonies shown in Figures 2, 3, and 4 are calculated as Lac^+ ^colonies per million chloramphenicol-resistant viable cells plated (or per million viable cells, in the cases of the single copy strains).

### Expansion test

Revertant colonies appearing on the M9 lactose plates were streaked on M9 lactose plates, which were incubated at 37°C until visible colonies formed. Heavy inocula constituting the bulk of the growth from the streaks were then scraped from the plate and grown to saturation in 5 ml M9 0.1% lactose minimal medium at 37°C. (Some of the revertant colonies tested, in the wild type background only, were inoculated directly from the selection plates into liquid lactose minimal medium; but all revertants in the wild type background tested positive for expanded arrays, regardless of the variation in culture methods). DNA was extracted by the use of a procedure involving freezing and thawing, lysozyme digestion, extraction with a phenol/chloroform/isoamyl alcohol mixture, extraction with ether, and precipitation with ethanol [30]. Portions were digested with EcoR1 and RNase, and subjected to electrophoresis in an agarose gel, followed by ethidium bromide staining. For quantitation of the repeat-specific bands, standards consisting of HindIII-digested phage lambda DNA of known concentration were included in the gel, in separate lanes. Total DNA in the sample was quantitated by spotting RNase-treated samples on an agarose slab containing ethidium bromide at 1 μg/ml, along with standards of known concentration. Band and spot intensities were measured by the use of digital photography and Kodak 1D software.

### Survival test

Mutations to be tested for their effects on survival on lactose minimal medium were crossed into a strain bearing a deletion of the *lac *operon. Cultures were grown to saturation in M9 0.1% glycerol as in the Lac^+ ^reversion test, and deposited on the surfaces of 0.6 ml M9 lactose agar plugs at the bottom of 12 × 75 mm plastic tubes, at approximately the same plating density (relative to volume of medium) as the bacteria in the Lac^+ ^reversion test. The tubes were incubated at 37°C. Cells were suspended by vortexing in 2 ml of buffer, and titered on M9 glucose plates, on days 0 and 7. The ratio of titer on day 7 to titer on day 0 for the wild type control was 0.83 ± 0.12 (mean ± standard error from six measurements).

## Abbreviations

Kbp: kilobasepair.
